# Evaluation of a Direct-from-Blood Molecular Diagnostic Platform for Pathogen Detection in Critically Ill Patients: A Prospective Observational Cohort Study

**DOI:** 10.1093/ofid/ofag486

**Published:** 2026-08-13

**Authors:** Yupeng Liu, Helen Donnelly, Alvaro Donayre, Erin Korth, Bridget Giblin, Francisco Martinez, Katie Clepp, Richard G Wunderink, Chiagozie Pickens

**Affiliations:** Department of Internal Medicine, Northwestern University Feinberg School of Medicine, Chicago, Illinois, USA; Division of Pulmonary and Critical Care Medicine, Northwestern University Feinberg School of Medicine, Chicago, Illinois, USA; Division of Pulmonary and Critical Care Medicine, Northwestern University Feinberg School of Medicine, Chicago, Illinois, USA; Division of Pulmonary and Critical Care Medicine, Northwestern University Feinberg School of Medicine, Chicago, Illinois, USA; Division of Pulmonary and Critical Care Medicine, Northwestern University Feinberg School of Medicine, Chicago, Illinois, USA; Division of Pulmonary and Critical Care Medicine, Northwestern University Feinberg School of Medicine, Chicago, Illinois, USA; Division of Pulmonary and Critical Care Medicine, Northwestern University Feinberg School of Medicine, Chicago, Illinois, USA; Division of Pulmonary and Critical Care Medicine, Northwestern University Feinberg School of Medicine, Chicago, Illinois, USA; Division of Pulmonary and Critical Care Medicine, Northwestern University Feinberg School of Medicine, Chicago, Illinois, USA

**Keywords:** bloodstream infection, diagnostic, molecular diagnostic, PCR, sepsis

## Abstract

**Rationale:**

Defining the microbial etiology of sepsis is difficult but has important implications for antibiotic stewardship and prognosis. We explore the diagnostic yield of a novel, direct-from-blood multiplex polymerase chain reaction in intensive care unit patients with suspected sepsis.

**Methods:**

From March 2022 to March 2024, patients were included if the clinical team ordered a blood culture. In study participants, an additional 6 mL of whole blood was collected by the phlebotomist. Samples were analyzed by the RaPID/BSI platform, a polymerase chain reaction-based assay that detects 19 bacterial and fungal species direct from whole blood.

**Results:**

A total of 165 samples were included in the final cohort. Of these, 27 patients had a positive RaPID/BSI result and 8 patients had positive blood cultures with 1 pathogen not included in the RaPID/BSI panel. Of the 27 patients with positive RaPID/BSI results, 5 patients also had positive blood cultures. Pathogen identification from RaPID/BSI and blood cultures were concordant for 4 of 5 of these pathogens. Twenty-five of the 27 RaPID/BSI positive patients had clinically defined infections. Eighteen patients of the 27 RaPID/BSI-positive patients had additional positive microbial culture data that corroborated pathogen identification. Of the 27 patients with bloodstream infections detected by RaPID/BSI and/or blood cultures, 20 had received antibiotics in the 48 hours before their blood draw. The RaPID/BSI assay identified the pathogen in all 20 of these patients, whereas only 4 of these 20 patients had positive blood cultures.

**Conclusions:**

The diagnostic yield of a direct-from-blood molecular diagnostic assay for the detection of bloodstream infection was 15.2% compared to 4.3% from blood cultures, providing higher detection rate especially in the setting of recent antibiotic administration.

## Teaser

The Resistance and Pathogen ID (RaPID) molecular diagnostic platform offers substantial additional diagnostic yield over conventional blood cultures while maintaining high specificity for clinically relevant infections.

Infection is a leading cause of death in critically ill patients and is associated with substantial morbidity and healthcare expenditures [1–3]. Early initiation of pathogen-directed antimicrobial therapy is crucial to reduce mortality and morbidity from infection [4, 5]. However, early and accurate culture-based pathogen identification, the current standard, remains challenging. While obtaining specimens directly from the site of infection (abscesses, bronchoalveolar lavage, biliary fluid, etc.) may have high diagnostic yield, these specimens often require invasive procedures and carry contamination risks that confound diagnostic yields [6]. Blood cultures are an alternative and complementary approach to detecting either primary or secondary bloodstream infections but are very often negative in critically ill patients, even in the setting of an established infection [7, 8]. A major factor contributing to culture negativity is administration of concurrent antimicrobial therapy, which is common among critically ill patients. Prior antimicrobial exposure is known to inhibit microbial growth in culture-based systems, often reducing diagnostic sensitivity by as much as 50% [9, 10].

Molecular diagnostics may address some of the limitations of culture-based diagnostics, and their application to blood samples is an area of active research. Most existing platforms require growth of an organism from blood culture prior to nucleic acid amplification-based species identification [11]. Other platforms such as those that use metagenomic DNA sequencing can detect microbial genetic material directly from blood but often cannot differentiate active from resolved infection, colonization, or contaminants [12–14]. In particular, the detection of commensal and contaminant organisms limits specificity, and correlation with additional data points is necessary to determine the clinical relevance of findings [15–18]. A systematic review and meta-analysis of 11 rapid molecular assays versus blood culture for bloodstream infections reported a pooled specificity of 0.86 and sensitivity of 0.66 by patient, indicating a need for higher performance molecular diagnostics for bloodstream infection [19]. In addition, the potential clinical impact of these platforms, particularly in the intensive care unit (ICU) where bloodstream infection is common, remains unclear.

Existing culture-independent molecular diagnostics are limited both by poor sensitivity, resulting in a significant portion of infections remaining undetected, and the insufficient specificity which complicates antimicrobial stewardship through potential over-diagnosis. In particular, the occurrence of positive molecular findings alongside negative blood cultures can create a potential credibility gap regarding the clinical relevance of the result. There is an increasing recognition of the need for high-performance, culture-independent molecular tools to provide more reliable pathogen identification in bloodstream infections [20, 21].

We therefore conducted a study of the Resistance and Pathogen ID (RaPID) molecular diagnostic platform, which incorporates several unique features designed to overcome limitations of other culture-independent molecular diagnostics. These include capacity to assay larger volumes of blood to improve diagnostic sensitivity and crucially, the ability to selectively detect the genetic material of only viable microbial cells rather than residual cell-free DNA. The RaPID/BSI platform identifies 19 clinically relevant pathogens directly from blood. Given the attributes of the platform, we hypothesized that RaPID/BSI would increase diagnostic yield in medical ICU patients who had blood cultures obtained for suspected infection.

## METHODS

### Patient Cohort

This single-center, prospective, observational cohort study was conducted in the Medical ICU at Northwestern Memorial Hospital. Blood samples were collected and analyzed with the RaPID/BSI platform prospectively (IRB #STU00216315) from March 2022 to March 2024, with the goal of enrolling 200 participants. Participants were screened and approached for study inclusion if they were 18 years of age or older and considered “at risk” for an invasive microbial infection based on diagnostic blood cultures ordered by the primary clinical team.

### Sample Collection

Eligible participants had a blood sample collected in 6-mL K2EDTA specimen tubes from the same venipuncture during routine collection of blood cultures. Specimen tubes were transported to the clinical laboratory with the blood culture bottles, where the specimen drawn for the RaPID/BSI test was placed in 2–8°C storage. The deidentified sample was labeled with a unique study identifier and shipped at 2–8°C to the study sponsor (Helixbind; Boxborough, MA) within 24 hours of collection. RaPID/BSI results were not communicated to the treating clinical team and all further diagnostic testing was at the discretion of the primary treating team. After testing, bidirectional sequencing of residual genetic material from the sample was performed to confirm all positive RaPID/BSI results. All data remained blinded until the end of study. After reviewing RaPID/BSI results, a separate protocol was designed and approved for retrospective clinical data collection to allow for comparison of the RaPID/BSI results to complete clinical diagnostic test results (IRB #STU00223594).

### RaPID Platform and RaPID/BSI

RaPID is a sample-to-answer molecular diagnostic platform [9]. RaPID/BSI, the first assay to be developed for the platform, identifies 19 prominent bacteria and fungi directly from blood with single CFU/mL sensitivity in roughly 5 hours. These include a broad panel of Gram-positive bacteria (*Staphylococcus aureus, Enterococcus faecalis, E. faecium, Streptococcus agalactiae, S. pyogenes,* and *S. pneumoniae*), Gram-negative bacteria (*Escherichia coli, Klebsiella aerogenes, Enterobacter cloacae, K. oxytoca, K. pneumoniae, Serratia marcescens, Pseudomonas aeruginosa,* and *Acinetobacter baumannii*), and yeast species (*Candida albicans, C. glabrata, C. krusei, C. parapsilosis,* and *C. tropicalis*). Because the assay is independent of culture growth, results are less impacted by previous or ongoing antimicrobial treatment.

The RaPID/BSI assay begins with a proprietary selective lysis that preserves viable microbial cells while removing released human DNA and microbial cell-free DNA (cfDNA). This elimination of human genetic material allows the platform to process multimillimeter volumes of blood, providing a mechanism to detect pathogens at extremely low titers. Additionally, the removal of cfDNA ensures that the assay targets only genetic material intact pathogens. Following selective lysis, viable microbial cells are chemically lysed to preserve high-molecular-weight microbial genomic DNA. This high-quality DNA is isolated and amplified using full-length 16S/18S rDNA primers to generate long amplicons. These long amplicons facilitate the design of comprehensive test menus and serve to distinguish the target material from degraded microbial DNA contamination. Target identification is completed using species-specific, artificial double-stranded DNA invading probes. Through a duplex-DNA invasion reaction, these probes identify microbial targets with single-base precision, providing high-confidence results even at minimal microbial loads. Use of artificial DNA probes ensures that only viable, actively proliferating pathogens are detected. The RaPID/BSI assay for bloodstream infections has been previously tested in comparison with follow-up blood cultures using retrospective samples and has demonstrated >94% sensitivity and >99% specificity across the assay menu [9, 10].

### Primary Outcome

The primary outcome of this study was the additive diagnostic yield of the RaPID/BSI platform in critically ill patients who had routine standard-of-care blood cultures obtained. The additive diagnostic yield is defined as the number of cases in which only RaPID/BSI was positive minus the number of cases in which only blood cultures were positive out of the total number of cases. In the retrospective study, we expanded the evaluation of diagnostic results for infection to include additional sources of infection, consistent with standard guidelines, and prior/subsequent blood culture results in addition to that concurrent with the RaPID/BSI assay [22].

### Clinical Adjudication and Statistical Analysis

Demographic, clinical, antibiotic, and microbiologic data were abstracted from the electronic medical record by the research team and entered into a standardized case report form in RedCap. Chart review was performed by 3 investigators (Y.L., C.P., R.G.W.) to determine whether each patient had a clinical picture consistent with infection, the site of infection, and results of diagnostic tests for infection. In patients without infection, the investigator documented the noninfectious etiology of inflammation. Continuous variables were reported as medians with the first and third interquartile ranges in brackets and compared using the Kruskal-Wallis tests. Adjustment for multiple comparisons was performed using the Benjamini-Hochberg method. Categorical variables were compared using Pearson's chi-squared test or Fisher's exact test.

## RESULTS

**Figure 1.**
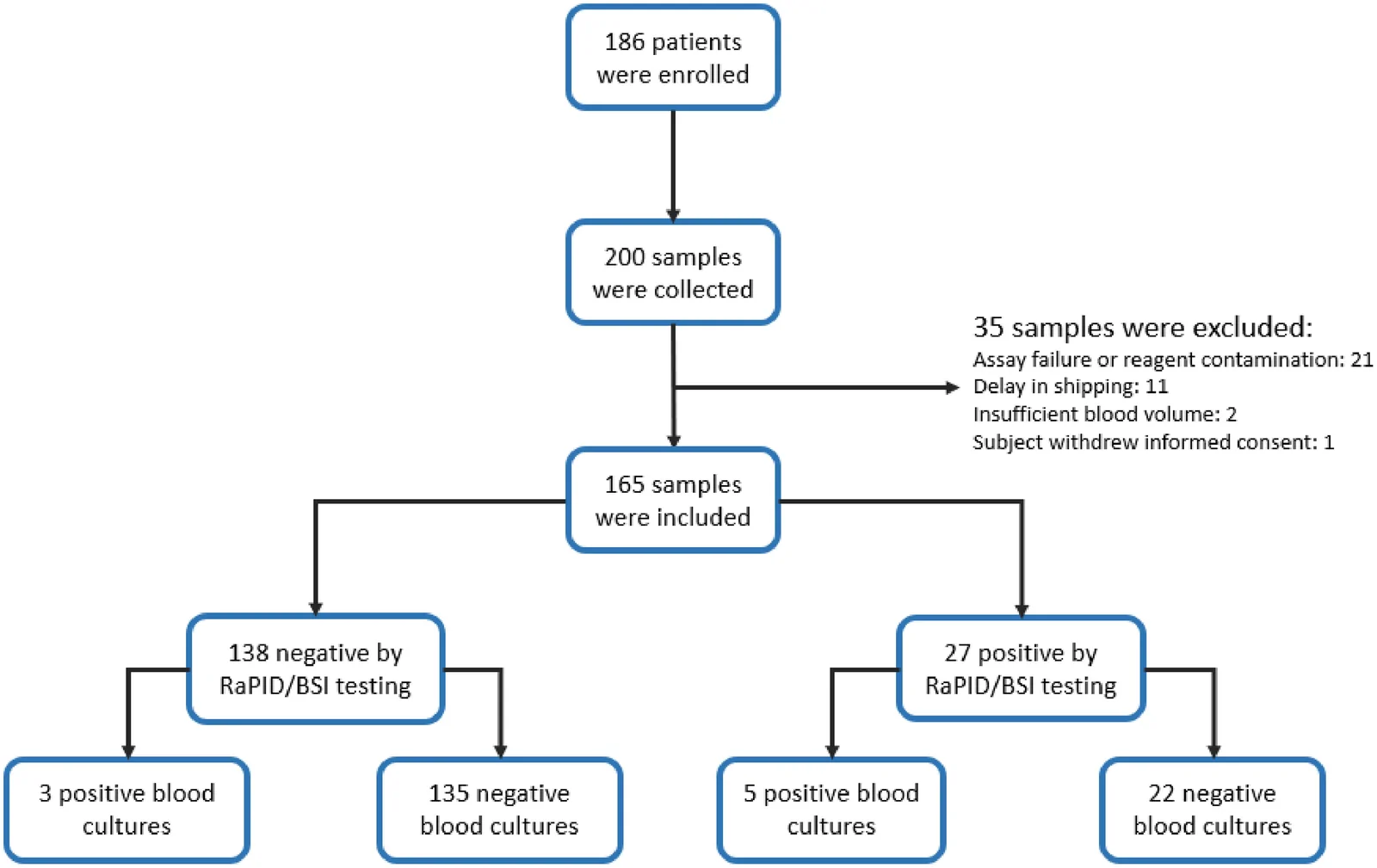
STARD diagram depicting study enrollment, reasons for subject exclusion, and diagnostic classification of included subjects.

Two hundred participants were enrolled in this study. One participant withdrew informed consent, and the patient's data and results were removed. A total of 17% (34/199) of samples were excluded because of delays with shipping, insufficient blood volume, or reagent contamination during sample testing (Figure 1). The remaining 165 samples were included in this analysis. Patient characteristics are presented in Table 1 including key clinical comorbidities that significantly increased clinical risk for infection, such as malignancy, organ transplant, cirrhosis, and immunosuppression. The presence of indwelling catheters, prostheses, and other devices were also included as risk factors. Discharge disposition was provided if information was available.

**Table 1.** Baseline Demographics, Patient Characteristics, and Discharge Disposition of the Study Cohort

Characteristic	N	N = 165^a^
Age, years	164	57 (16)
Gender	165	…
Female	…	70 (42%)
Male	…	95 (58%)
Ethnicity	165	…
Hispanic or Latino	…	20 (12%)
Not Hispanic or Latino	…	134 (81%)
Unknown	…	11 (6.7%)
Diabetes	153	23 (15%)
Hematologic malignancy	153	17 (11%)
Solid malignancy	153	8 (5.2%)
Organ transplant	153	16 (10%)
Liver cirrhosis	153	9 (5.9%)
Neutropenia	153	3 (2.0%)
Neutropenia within 2 weeks before sample collection	153	6 (3.9%)
Other immunosuppressive condition	153	3 (2.0%)
Peripherally inserted central catheter	153	21 (14%)
Nontunneled central line	153	32 (21%)
Tunneled line	153	13 (8.5%)
Pacemaker	153	2 (1.3%)
Joint prosthesis	153	1 (0.7%)
Other indwelling device	153	28 (18%)
Discharge disposition from hospital	97	…
Acute inpatient rehabilitation	…	14 (14%)
Death	…	36 (37%)
Home	…	24 (25%)
Long-term acute care hospital	…	7 (7.2%)
Skilled nursing facility	…	13 (13%)
Transfer to an outside hospital	…	3 (3.1%)

^a^Mean (SD); n (%).

Eight samples were positive on routine blood cultures. Analyses of RaPID/BSI results and infection status are summarized in Table 2, which compares RaPID/BSI result and the presence of bloodstream infection at time of sample. One blood culture pathogen was not on the RaPID/BSI panel (viridans group *Streptococcus*) and was omitted from performance assessment of the RaPID/BSI in Table 2.

**Table 2.** RaPID/BSI Result and Presence of Bloodstream Infection, Defined as a Positive Pathogen Detection on RaPID/BSI or Blood Culture and a Clinically Defined Infection

	RaPID/BSI Positive	RaPID/BSI Negative	Total
Bloodstream infection at time of RaPID/BSI draw	25	1	26
No bloodstream infection at time of RaPID/BSI draw	2	136	138
Total	27	137	164

Twenty-seven of 165 patients had a positive RaPID/BSI result. Table 3 displays their clinical characteristics and microbiologic culture data. The type of active clinical infection(s) present at time of RaPID/BSI sample collection is specified, as well as the presence of additional culture data from blood and other sample types. Bidirectional rDNA sequencing was performed on 25 and confirmed detections in all, with insufficient material available for testing in the remaining 2 cases. Because of high sequence homology within the Enterobacterales order, rDNA sequencing is often unable to provide definitive species level differentiation between certain closely related species, such as *Klebsiella* and *Enterobacter*.

**Table 3.** Clinical Characteristics and Microbiologic Culture Data in the RaPID/BSI Positive Cohort

Record	RaPID/BSI	Bidirectional DNA sequencing	Clinical infection(s)	Microbiologic concordance	Blood cultures	Antibiotics administered	Prior blood cultures	Other microbiologic data
6	Non-*E. coli Enterobacterales*	*Enterobacter* spp./*Klebsiella* spp.	Septic arthritis of knee	Concordant	No growth	Yes	*K. pneumoniae*	Synovial fluid with *K. pneumoniae*
19	*E. faecium*	E. faecium	GI/intra-abdominal	No supporting culture data	No growth	No	…	…
21	Non-*E. coli Enterobacterales*	*Enterobacter* spp./*Klebsiella* spp.	No documented infection site	No supporting culture data	No growth	No	…	…
28	Non-*E. coli Enterobacterales*	*Enterobacter* spp./*Klebsiella* spp.	Pneumonia	Discordant	Staphylococcus aureus	No	*S. aureus*	…
34	*S. agalactiae/S. pyogenes*	*S. agalactiae*	Wound/soft-tissue GU	Discordant	No growth	Yes	*S. aureus*	…
36	Non-*E. coli Enterobacterales*	*Pantoea* spp.	GI/intra-abdominal	No supporting culture data	No growth	Yes	…	…
43	*S. pneumoniae*	*S. pneumoniae*	Pneumonia	Concordant	No growth	Yes	*S. pneumoniae*	Streptococcal urinary antigen
57A	Non-*E. coli Enterobacterales*	*Enterobacter* spp./*Klebsiella* spp.	GI/intra-abdominal	Concordant	*E. cloacae*	No	…	…
57B^a^	Non-*E. coli Enterobacterales*	*Enterobacter* spp./*Klebsiella* spp.	Pneumonia	Concordant	No growth	Yes	*E. cloacae*	…
59	*S. aureus*	*S. aureus*	CLABSI	Concordant	No growth	No	*S. aureus*	…
62	*E. faecalis*	*E. faecalis*	Pneumonia Empyema	Discordant	No growth	Yes	…	Empyema aspiration with Ureaplasma
63	*S. aureus*	*S. aureus*	GI/intra-abdominal	Concordant	No growth	Yes	…	Peritoneal fluid with *S. aureus*
91	*S. aureus; S. agalactiae/S. pyogenes*	*S. aureus*; *S. agalactiae*	Pneumonia Wound/soft-tissue	Concordant	No growth	Yes	*S. agalactiae*	NBBAL culture with *S. aureus* PCR with *S. aureus* and *K. pneumoniae*
93	*S. agalactiae/S. pyogenes*	*S. pyogenes*	Wound/soft-tissue	Concordant	No growth	Yes	*S. pyogenes*	…
94	*S. aureus*	Not performed	No documented infection site	No supporting culture data	No growth	Yes	…	…
96	*E. coli*	*E. coli*	GU	Concordant	No growth	Yes	*E. coli*	Urine culture with *E. coli*
97	*S. agalactiae/S. pyogenes*	*S. agalactiae*	Pneumonia Endocarditis	Concordant	No growth	Yes	*S. agalactiae*	…
98	*E. faecalis*	*E. faecalis*	CLABSI	Concordant	*E. faecalis*	Yes	*E. faecalis*	…
99	*Candida* spp.	*Candida* spp.	Pneumonia CLABSI GU	Concordant	*C. tropicalis*	Yes	*C. tropicalis*	Urine culture BAL with *C. tropicalis*
100	*S. agalactiae/S. pyogenes*	*S. agalactiae*	Endocarditis	Concordant	No growth	Yes	*S. agalactiae*	…
102	*S. aureus*	Not performed	Pneumonia	Concordant	No growth	Yes	…	NBBAL with *S. aureus* and PCR
109	*S. aureus*	*S. aureus*	Pneumonia Wound/soft-tissue	Discordant	No growth	No	…	BAL with *E. cloacae* and *E. faecalis*, PCR with *E. cloacae* Groin wound culture with *E. cloacae, E. faecium*
125	*S. aureus*	*S. aureus*	Wound/soft-tissue Endocarditis GU	Concordant	No growth	Yes	…	Urine culture with *S. aureus* Wound PCR with *S. aureus*
149	Non-*E. coli Enterobacterales*	*Enterobacter* spp./*Klebsiella* spp.	Pneumonia	Concordant	No growth	Yes	…	Sputum culture with *K. pneumoniae*
155	*E. coli; E. faecium*	*E. coli*; *E. faecium*	GI/intra-abdominal	Concordant	*E. faecium*	Yes	*E. coli E. faecium*	…
157	Non-*E. coli Enterobacterales*	*Enterobacter* spp./*Klebsiella* spp.	Pneumonia GI/intra-abdominal	No supporting culture data	No growth	Yes	…	…
169	*S. agalactiae/S. pyogenes*	*S. pyogenes*	Pneumonia GI/intra-abdominal	Concordant	No growth	Yes	*S. pyogenes*	Toxigenic *E. coli* on GI panel

Abbreviations: CLABSI, central line-associated bloodstream infection; GI, gastrointestinal; GU, genitourinary; NBBAL, nonbronchoscopic bronchoalveolar lavage; PCR, polymerase chain reaction.

Microbiologic concordance indicates whether the RaPID/BSI result was concordant or discordant with available culture data. Antibiotic administration indicates whether patients received antibiotic therapy within 48 hours before blood draw. Prior blood cultures included positive blood cultures within the 7 days before RaPID/BSI sample collection.

^a^Samples 57B drawn from same subject 5 days later.

For patients with positive RaPID/BSI results, 19% (5/27) had positive blood cultures from the same blood draw as the RaPID/BSI collection. Pathogen identification from RaPID/BSI and blood cultures were concordant for 4 of these pathogens. Three patients with concordant RaPID/BSI and blood culture results also had additional microbiological culture data that further confirmed the specific pathogen identification. One patient had a RaPID/BSI detection of non–*E. coli Enterobacterales* with discordant blood culture identification of *S. aureus* without additional culture data available.

A total of 93% (25/27) of patients with a positive RaPID/BSI had clinically defined infections. One patient with a positive RaPID/BSI for non-*E. coli Enterobacterales* had no evidence of active infection. Another patient underwent infectious evaluation for acute respiratory failure and had RaPID/BSI positive for *S. aureus* with negative blood cultures, and after clinical adjudication was determined to have pulmonary leukostasis from acute myeloid leukemia as a cause of respiratory failure rather than infection. Taken together, the diagnostic yield of RaPID/BSI was 15.2% (25/164; 95% confidence interval [CI], 9.7–20.4) compared to the 4.3% (7/164; 95% CI, 1.2–7.4) diagnostic yield of blood culture. The additive diagnostic yield was 12% (18/164; 95% CI, 6.2–15.8).

### Evaluation of the RaPID/BSI Negative Cohort

Three of 138 patients with a negative RaPID/BSI result had a positive concurrent blood culture. Two blood culture pathogens, *Corynebacterium striatum* and *Staphylococcus epidermis* (regarded as contaminant and not treated), were not on the RaPID/BSI panel. The third patient was diagnosed with pneumonia and grew *Acinetobacter baumannii* in 1 of 2 blood culture sets but not from a bronchoalveolar lavage fluid culture or polymerase chain reaction (PCR). This blood culture was considered a contaminant by both the primary and infectious disease consultant teams. Although the patient received empirical treatment because of clinical instability, the negative RaPID/BSI result was concordant with the clinical determination that the single positive blood culture bottle did not represent a true bloodstream infection.

### Concordance With Microbiological Culture Data

Of 25 patients with positive RaPID/BSI and clinically defined infections, 18 (72%) had additional culture data that corroborated the identified pathogens, with some patients having multiple confirmatory cultures from additional microbiological samples. Sample sources included blood (6), bronchoalveolar lavage (4), wound or soft tissue (4), urine (4), sputum (1), and peritoneal fluid (1). Notably, 2 patients had multiple species identified by the RaPID/BSI assay with each species corroborated by microbiologic cultures from elsewhere in the body. One patient with pneumonia and a skin and soft-tissue infection had RaPID/BSI positive for both *S. aureus* and *S. agalactiae/S. pyogenes*. Additional workup revealed *S. agalactiae* from blood cultures and bronchoalveolar lavage culture and PCR both positive for *S. aureus*. The second patient, diagnosed with an intraabdominal infection, was RaPID/BSI positive for both *E. coli* and *E. faecium,* consistent with blood culture results within the prior 7 days.

### RaPID/BSI Detects Infectious Pathogens in Blood Culture-negative Patients After Antibiotic Administration

Of the 27 patients with bloodstream infections detected by RaPID/BSI and/or blood cultures, 20 (74%) received antibiotics in the 48 hours before their blood draw. The RaPID/BSI assay identified the pathogen in all 20 of these patients (100%), whereas only 4 patients had positive blood cultures (20%).

## DISCUSSION

In this prospective observational study, we demonstrate a significant increase in diagnostic yield of the RaPID/BSI platform in critically ill patients. Importantly, nearly all patients with a positive RaPID/BSI had clinically defined infections, with the majority demonstrating species-level concordance with either blood cultures or cultures of fluid or tissue samples from elsewhere in the body. The substantive additive diagnostic yield, especially in patients with antibiotic exposure, combined with low rates of false positivity, are paramount for direct-from-blood, culture-independent molecular diagnostics. RaPID/BSI is not restricted to patients who already have positive blood cultures; therefore, the RaPID/BSI assay provides valuable additional microbiologic data from a routine blood draw in patients with negative blood cultures but high suspicion for infection, potentially optimizing clinical management.

A major limitation in the diagnostic yield of blood cultures for critically ill patients is reduced sensitivity following antibiotic initiation [23, 24]. In our cohort, the RaPID/BSI assay demonstrated high efficacy in identifying bacterial pathogens among patients with negative blood cultures after antibiotic administration up to 48 hours before the blood draw. This finding is consistent with results of in vitro experiments wherein the addition of antibiotic cocktails at supraphysiological concentrations to culture mediums with preselected microorganisms had no impact on RaPID/BSI sensitivity [9]. Because performance is less affected by concurrent antibiotics, RaPID/BSI may offer the potential to circumvent delays in antibiotic initiation while awaiting blood culture collection as yield may be relatively preserved compared to blood cultures, although further studies would be required to establish this finding.

An intrinsic concern with molecular diagnostic tests is the possibility of detecting microbial DNA from sources other than infection, such as contaminant, colonization, or previous resolved infection. The low false positivity rate demonstrated by RaPID/BSI in our study (2/138 or 1.4%) suggests that the assay's initial step of removing cell-free DNA from nonviable cells substantially reduces this likelihood. One patient's false-positive result with detection of *S. aureus* likely resulted from skin contamination. The other patient's detection of non-*E. coli Enterobacterales* may reflect contamination of assay reagents or at the site of testing. Nonetheless, RaPID/BSI's false-positive rate was lower than the estimated 20% to 50% false-positive rate of blood cultures in hospitalized adult patients, which reflects an approximate contamination rate of 2% to 3% among all blood cultures [25]. This reduction is likely driven by a combination of the assay's technical attributes and the logistical workflow, in which blood for RaPID/BSI was collected after the initial volume for routine cultures. Ultimately, these exceptionally low false-positive rates compare favorably against other culture-independent molecular diagnostics, which are often limited by poor specificity in the setting of bloodstream infections [7].

One of the most distinctive aspects of the RaPID platform is the detection of only viable microbes. In addition to minimizing false-positive rates, this unique aspect has important clinical implications. Of positive RaPID/BSI samples with negative concomitant blood cultures after antibiotic administration, 10/18 (55.6%) were blood culture-positive in the previous 7 days. Presence of viable bacteria after initiation of antibiotic treatment may indicate inadequate source control or ineffective antimicrobial therapy, even if conventional blood cultures fail to identify pathogen presence. The higher accuracy of RaPID/BSI to detect lack of pathogen clearance after treatment initiation than blood cultures further expands its potential clinical utility. These preliminary results for RaPID/BSI test suggest this unique molecular assay offers substantial additional diagnostic yield over conventional blood cultures while maintaining high specificity for clinically relevant infections.

Key limitations of this study include its single-center design, which restricts the generalizability of study results to other healthcare systems and geographic regions. The necessity of shipping samples to the study sponsor for processing introduced preanalytical variability. In real-world practice, the absence of an onsite RaPID/BSI analyzer reduces the clinical advantages of rapid detection of viable pathogens. Similarly, the practical utility of RaPID/BSI pathogen detection must be investigated with specific clinical endpoints such as changes in antibiotic management and clinical trajectory. Further studies are necessary to validate the operating characteristics of the RaPID/BSI assay and define its role within an evolving diagnostic paradigm. Once commercially available, prospective interventional trials are necessary to determine the assay's true clinical utility.

## References

[1] Vincent JL, Sakr Y, Singer M, et al Prevalence and outcomes of infection among patients in intensive care units in 2017. JAMA 2020; 323:1478–87. 32207816 10.1001/jama.2020.2717 PMC7093816

[2] Cassini A, Plachouras D, Eckmanns T, et al Burden of six healthcare-associated infections on European population health: estimating incidence-based disability-adjusted life years through a population prevalence-based modelling study. PLoS Med 2016; 13:e1002150. 27755545 10.1371/journal.pmed.1002150 PMC5068791

[3] Zimlichman E, Henderson D, Tamir O, et al Health care–associated infections: a meta-analysis of costs and financial impact on the US health care system. JAMA Intern Med 2013; 173:2039–46. 23999949 10.1001/jamainternmed.2013.9763

[4] Seymour CW, Gesten F, Prescott HC, et al Time to treatment and mortality during mandated emergency care for sepsis. N Engl J Med 2017; 376:2235–44. 28528569 10.1056/NEJMoa1703058 PMC5538258

[5] Ferrer R, Martin-Loeches I, Phillips G, et al Empiric antibiotic treatment reduces mortality in severe sepsis and septic shock from the first hour: results from a guideline-based performance improvement program. Crit Care Med 2014; 42:1749. 24717459 10.1097/CCM.0000000000000330

[6] Miller JM, Binnicker MJ, Campbell S, et al A guide to utilization of the microbiology laboratory for diagnosis of infectious diseases: 2018 update by the Infectious Diseases Society of America and the American Society for Microbiology. Clin Infect Dis 2018; 67:e1–94. 29955859 10.1093/cid/ciy381 PMC7108105

[7] Fabre V, Hsu YJ, Carroll KC, et al Blood culture use in medical and surgical intensive care units and wards. JAMA Netw Open 2025; 8:e2454738. 39813030 10.1001/jamanetworkopen.2024.54738 PMC11736503

[8] Fabre V, Klein E, Salinas AB, et al A diagnostic stewardship intervention to improve blood culture use among adult nonneutropenic inpatients: the DISTRIBUTE study. J Clin Microbiol 2020; 58:e01053–20. 32759354 10.1128/JCM.01053-20 PMC7512168

[9] Iyer V, Castro D, Malla B, et al Culture-independent identification of bloodstream infections from whole blood: prospective evaluation in specimens of known infection status. J Clin Microbiol 2024; 62:e0149823. 38315022 10.1128/jcm.01498-23 PMC10935643

[10] Nölling J, Rapireddy S, Amburg JI, et al Duplex DNA-invading γ-modified peptide nucleic acids enable rapid identification of bloodstream infections in whole blood. mBio 2016; 7:e00345–16. 27094328 10.1128/mBio.00345-16 PMC4850259

[11] Rhoads DD, Pournaras S, Leber A, et al Multicenter evaluation of the BIOFIRE blood culture identification 2 panel for detection of bacteria, yeasts, and antimicrobial resistance genes in positive blood culture samples. J Clin Microbiol 2023; 61:e0189122. 37227281 10.1128/jcm.01891-22 PMC10281132

[12] Ma R, Yin Y, Zhang JP, et al Metagenomic next-generation sequencing compared with blood culture as first-line diagnostic method for bloodstream infection in hematologic patients with febrile neutropenia: a multicenter, prospective study. Open Forum Infect Dis 2025; 12:ofaf288. 40453877 10.1093/ofid/ofaf288 PMC12125677

[13] Qian M, Li C, Zhang M, et al Blood metagenomics next-generation sequencing has advantages in detecting difficult-to-cultivate pathogens, and mixed infections: results from a real-world cohort. Front Cell Infect Microbiol 2023; 13:1268281. 38188631 10.3389/fcimb.2023.1268281 PMC10768086

[14] Nielsen ME, Søgaard KK, Karst SM, Krarup AL, Albertsen M, Nielsen HL. Application of rapid Nanopore metagenomic cell-free DNA sequencing to diagnose bloodstream infections: a prospective observational study. Microbiol Spectr 2025; 13:e0329524. 40135889 10.1128/spectrum.03295-24 PMC12054037

[15] Miller S, Chiu C. The role of metagenomics and next-generation sequencing in infectious disease diagnosis. Clin Chem 2021; 68:115–24. 34969106 10.1093/clinchem/hvab173

[16] Simner PJ, Miller S, Carroll KC. Understanding the promises and hurdles of metagenomic next-generation sequencing as a diagnostic tool for infectious diseases. Clin Infect Dis 2018; 66:778–88. 29040428 10.1093/cid/cix881 PMC7108102

[17] Miao Q, Ma Y, Wang Q, et al Microbiological diagnostic performance of metagenomic next-generation sequencing when applied to clinical practice. Clin Infect Dis 2018; 67:S231–40. 30423048 10.1093/cid/ciy693

[18] Liu J, Zhang Q, Dong YQ, Yin J, Qiu YQ. Diagnostic accuracy of metagenomic next-generation sequencing in diagnosing infectious diseases: a meta-analysis. Sci Rep 2022; 12:21032. 36470909 10.1038/s41598-022-25314-y PMC9723114

[19] Rapszky GA, Do To UN, Kiss VE, et al Rapid molecular assays versus blood culture for bloodstream infections: a systematic review and meta-analysis. eClinicalMedicine 2025;79:103028. 39968206 10.1016/j.eclinm.2024.103028 PMC11833021

[20] Zhang Y, Hu A, Andini N, Yang S. A ‘culture’ shift: application of molecular techniques for diagnosing polymicrobial infections. Biotechnol Adv 2019; 37:476–90. 30797092 10.1016/j.biotechadv.2019.02.013 PMC6447436

[21] Makristathis A, Harrison N, Ratzinger F, et al Substantial diagnostic impact of blood culture independent molecular methods in bloodstream infections: superior performance of PCR/ESI-MS. Sci Rep 2018; 8:16024. 30375435 10.1038/s41598-018-34298-7 PMC6207717

[22] IDSA . New Fever in Critically Ill Patients. Available at: https://www.idsociety.org/practice-guideline/new-fever-in-critically-ill-patients/. Accessed May 14, 2026.

[23] Scheer CS, Fuchs C, Gründling M, et al Impact of antibiotic administration on blood culture positivity at the beginning of sepsis: a prospective clinical cohort study. Clin Microbiol Infect 2019; 25:326–31. 29879482 10.1016/j.cmi.2018.05.016

[24] Cheng MP, Stenstrom R, Paquette K, et al Blood culture results before and after antimicrobial administration in patients with severe manifestations of sepsis: a diagnostic study. Ann Intern Med 2019; 171:547–54. 31525774 10.7326/M19-1696

[25] Callado GY, Lin V, Thottacherry E, et al Diagnostic stewardship: a systematic review and meta-analysis of blood collection diversion devices used to reduce blood culture contamination and improve the accuracy of diagnosis in clinical settings. Open Forum Infect Dis 2023; 10:ofad433. 37674630 10.1093/ofid/ofad433 PMC10478151

